# Impact of High-Efficiency Dialysis Modalities on Interdialytic Blood Pressure Profiles: A Randomized Cross-Over Study

**DOI:** 10.3390/medicina61122077

**Published:** 2025-11-21

**Authors:** Jan Michał Biedunkiewicz, Agnieszka Zakrzewska, Katarzyna Jasiulewicz, Natalia Płonka, Bogdan Biedunkiewicz, Alicja Dębska-Ślizień, Leszek Tylicki

**Affiliations:** 1 Department of Anesthesiology and Intensive Therapy, Faculty of Medicine, Medical University of Gdańsk, Smoluchowskiego 17, 80-214 Gdańsk, Poland; 2 Department of Nephrology, Transplantology and Internal Diseases, Faculty of Medicine, Medical University of Gdańsk, Smoluchowskiego 17, 80-214 Gdańsk, Polandbogdan.biedunkiewicz@gumed.edu.pl (B.B.); adeb@gumed.edu.pl (A.D.-Ś.);

**Keywords:** HDx, HDF, hemodialysis, interdialytic blood pressure, home blood pressure monitoring

## Abstract

*Background and Objectives:* Interdialytic blood pressure (BP) better reflects volume status and cardiovascular risk in hemodialysis (HD) patients than peridialytic readings. High-efficiency dialysis techniques—online hemodiafiltration (HDF) in pre-, post-, and mixed-dilution modes, and expanded hemodialysis (HDx) with medium cut-off membranes—aim to improve solute clearance and hemodynamic stability. Their comparative impact on interdialytic BP control remains unclear. This randomized cross-over study compared interdialytic BP profiles across these modalities under standardized treatment conditions. *Materials and Methods*: Sixteen clinically stable adults with end-stage kidney disease sequentially underwent high-flux HD, HDx, and HDF in pre-, post-, and mixed-dilution configurations, each for one month. Dialysis prescriptions, dry weight, and antihypertensive therapy remained constant. Home BP was measured twice daily on non-dialysis days, yielding ~3600 observations. Systolic (SBP), diastolic (DBP), and mean arterial pressure (MAP) were analyzed by repeated-measures ANOVA with Bonferroni correction. *Results*: Significant differences were found among modalities for SBP (*p* = 0.009), DBP (*p* = 0.004), and MAP (*p* < 0.001). HDx achieved the lowest mean BP values—SBP 129 (95% CI 127–131) mmHg; DBP 74 (95% CI 73–75) mmHg; MAP 93 (95% CI 91–94) mmHg—significantly lower than high-flux HD and post-dilution HDF (*p* < 0.05). Differences versus pre- and mixed-HDF did not reach significance. *Conclusions*: HDx provided modest but consistent reductions in interdialytic BP compared with diffusive and convective high-efficiency modalities. Trial Registration: Ethics Committee of the Medical University of Gdańsk (NKBBN/479-759/2022).

## 1. Introduction

Hypertension remains one of the most prevalent and clinically significant complications in patients with end-stage renal disease (ESRD) receiving maintenance hemodialysis (HD). Interdialytic blood pressure (BP), particularly systolic blood pressure (SBP), is a strong predictor of cardiovascular morbidity and mortality in this population [1,2]. Although much attention has been directed toward intradialytic hypotension, accumulating evidence suggests that interdialytic BP patterns may better reflect volume status and cardiovascular risk than peridialytic readings alone [3,4]. Several high-efficiency dialysis modalities have been introduced to enhance solute clearance and potentially improve cardiovascular outcomes and hemodynamic stability. These include online hemodiafiltration (HDF) delivered in pre-, post-, or mixed-dilution modes, as well as expanded hemodialysis, which employs medium cut-off membranes to facilitate middle-molecule removal [5,6,7]. While these techniques have demonstrated improved biochemical profiles and survival benefits in selected trials [8,9,10,11], their comparative impact on interdialytic BP control has not been systematically characterized.

Several large randomized trials have investigated the long-term effects of HDF on survival and cardiovascular outcomes. The ESHOL trial demonstrated a significant reduction in all-cause mortality with high-volume post-dilution HDF compared to high-flux HD [8]. In contrast, the CONTRAST and Turkish OL-HDF studies reported neutral primary outcomes, although secondary analyses suggested improved outcomes with higher convection volumes [9,10]. The recent CONVINCE trial confirmed a survival benefit and better patient-reported outcomes with high-volume HDF, without notable differences in BP between treatment arms [11]. Expanded hemodialysis (HDx) has been proposed as a practical alternative to HDF, particularly in settings where access flow or infrastructure limits convective therapy delivery, but head-to-head comparisons remain sparse [6,12].

Blood pressure has typically been a secondary or exploratory endpoint in trials of dialysis modality, and few studies have directly compared multiple techniques beyond a standard HD control group. While several studies have compared subsets of high-efficiency dialysis modalities for example, HDx vs. HD, or mixed-, pre-, and post-dilution HDF none have yet included all HDF modes together with HDx and conventional HD in a single randomized cross-over cohort. Our work thus fills that crucial gap by directly comparing the full spectrum of modalities under identical treatment conditions.

Additionally no studies comparing different modalities have assessed BP exclusively during the interdialytic period, outside the controlled setting of dialysis sessions, where cardiovascular and volume-related stress may have significant prognostic value. Previous studies have explored the relationship between blood pressure dynamics and outcomes in hemodialysis patients. A meta-analysis by Li et al. demonstrated that greater interdialytic BP variability, rather than mean BP level, independently predicted higher cardiovascular and all-cause mortality in over 30,000 patients [13]. Conversely, a systematic review by Mostovaya et al. found that high-efficiency post-dilution HDF reduces all-cause and cardiovascular mortality compared with HD, with benefits proportional to achieved convection volume, yet without differences in BP between groups [14]. Together, these findings suggest that improved outcomes with convective modalities are not mediated through interdialytic BP control but through other mechanisms such as better solute clearance and volume stability.

Our study expands this evidence by directly comparing all modern high-efficiency dialysis techniques—including three HDF dilution modes and HDx—within the same patient cohort, under standardized treatment conditions and stable antihypertensive therapy. We conducted a randomized cross-over study analyzing interdialytic SBP, DBP, and mean arterial pressure (MAP) across five dialysis modalities: conventional high-flux HD, HDx, and HDF in pre-, post-, and mixed-dilution modes. BP was measured exclusively on non-dialysis days using standardized protocols, and dialysis prescriptions were held constant across modalities to isolate treatment-specific effects. This within-subject design allowed direct comparison of high-efficiency modalities under controlled conditions and provides new insight into their hemodynamic neutrality or potential superiority beyond the dialysis unit.

## 2. Materials and Methods

### 2.1. Study Design

This was a prospective, single-center, randomized cross-over study designed to evaluate interdialytic blood pressure profiles across five hemodialysis modalities. The modalities included conventional high-flux hemodialysis, hemodiafiltration in pre-, post-, and mixed-dilution configurations, and expanded hemodialysis using medium cut-off membranes each for one month. No washout period was introduced between phases. The primary endpoint was the comparison of home interdialytic SBP, DBP, and MAP across modalities. The study protocol was approved by the institutional ethics committee Ethical Committee at the Medical University of Gdańsk (no. NKBBN/479-759/2022; 18 November 2022), and written informed consent was obtained from all participants prior to enrollment.

### 2.2. Patients

Patient enrollment took place at the Dialysis Unit of the Department of Nephrology, Transplantology and Internal Medicine, Medical University of Gdańsk, during the final quarter of 2023. Eligible participants were adults undergoing maintenance hemodialysis three times per week for a minimum of six months. Additional inclusion criteria required a single-pool Kt/V_urea exceeding 1.2, body weight between 60 and 85 kg, and reliable vascular access permitting a blood flow rate above 350 mL/min, either via native fistula or central venous catheter.

Patients were excluded if they had an estimated life expectancy under six months, demonstrated poor adherence to dialysis procedures or medical recommendations, or presented with a high comorbidity burden (Age-Adjusted Charlson Comorbidity Index > 8). Further exclusion factors comprised recent hospitalization (within 30 days prior to study entry), active infection or malignancy, uremic neuropathy, pruritus, dialysis amyloidosis, or resistance to erythropoietin therapy. Individuals with intradialytic hemodynamic instability, uncontrolled hypertension (home BP > 140/90 mmHg), or excessive BP variability in pilot measurements (standard deviation of SBP or DBP > 10 mmHg) were likewise not considered for inclusion.

Eighteen clinically stable adults with end-stage renal disease (ESRD) undergoing maintenance HD were enrolled. Two patients did not complete the study—one underwent kidney transplantation and one died from a cause unrelated to the study. Thus, sixteen participants completed all five treatment phases and were included in the per-protocol analysis. Each patient underwent all five dialysis modalities in randomized order over a five-month period. Baseline clinical characteristics are summarized in Table 1; the mean age was 62.6 ± 12.7 years, 62.5% were male, and the leading causes of ESRD were diabetic and hypertensive nephropathy. The mean dialysis vintage was 75.8 ± 70.9 months, mean body-mass index 27.8 ± 5.6 kg/m^2^, and most patients had cardiovascular comorbidities—hypertension 93.8%, coronary artery disease 31.3%, and heart failure 12%. No protocol violations occurred during the study. No hospitalizations or severe intradialytic complications leading to discontinuation of dialysis session were recorded.

### 2.3. Dialysis Prescription and Equipment

Dialysis procedures were carried out on the Fresenius 5008 platform equipped with the AutoSub Plus module (Fresenius Medical Care, Bad Homburg, Germany). Conventional high-flux HD as well as HDF in pre-, post-, and mixed-dilution configurations utilized FX 100 dialyzers (membrane surface 2.2 m^2^; ultrafiltration coefficient 73 mL/h/mmHg; Fresenius Medical Care). Expanded hemodialysis (HDx) was performed using the Theranova 400 medium-cutoff dialyzer (surface 1.7 m^2^; UF coefficient 48 mL/h/mmHg; Baxter, Alliston, ON, Canada).

Each treatment lasted 240 min. Blood and dialysate flow rates were maintained at 300 mL/min and 500 mL/min, respectively, with dialysate temperature kept constant at 36.5 °C. Baseline dry weight was established by bioimpedance spectroscopy and remained unchanged during all study phases. The prescribed ultrafiltration volume was tailored individually to the patient’s interdialytic weight gain, fluid intake, and circuit priming volume. Ultrafiltration and sodium profiling features were intentionally disabled. The dialysate composition was standardized for all sessions: sodium 138–140 mmol/L, potassium 2.0–3.0 mmol/L, calcium 1.25–1.5 mmol/L, bicarbonate 32 mmol/L, magnesium 0.5 mmol/L, chloride 110 mmol/L, and glucose 1.0 g/L.

All patients received unfractionated heparin, administered as a bolus followed by continuous infusion per standard protocol. In HDF modes, sterile substitution fluid was generated online via ultrafiltration of ultrapure dialysate. Substitution rates and convection volumes were regulated automatically using the AutoSub Plus system, which adjusted flow settings based on pressure pulse attenuation and transmembrane pressure. The system continuously optimized filtration to reduce hemoconcentration and maximize convective efficiency. All treatment parameters, session time, fluid composition, flow rates, dialysate temperature, anticoagulation, and dry weight remained unchanged across modalities. Concomitant medications, including antihypertensive therapy, were maintained without adjustment throughout the study. Average total convection volume in liters for post-HDF, pre-HDF and mix-HDF were 20.68, 45.73 and 50.98 L, respectively. Detailed mean dialysis parameters during all study phases are presented in Table 2.

### 2.4. Outcomes

The main outcomes of our research protocol were the mean SBP, DBP and MAP measured on non-dialysis days for each dialysis modality. MAP was calculated as: MAP = DBP + 1/3 × (SBP − DBP). Home BP was measured according to current recommendations on every non-dialysis day throughout each treatment phase, yielding a total of approximately 70 readings per patient during one phase of the study. Measurements were taken twice daily: morning and evening, before meals, and before taking medications. During each measurement session, patients took two readings, at least one minute apart. Before the measurement, the patient remained in a quiet, seated position for 5 min. Measurements were taken with validated, automated upper arm blood pressure monitors, using the same device for each patient throughout all study phases. Average values from the entire monitoring period were used for analysis. Patients were educated by dialysis center staff and received written instructions on how to perform the measurements and record the results.

### 2.5. Statistics

Data from our previous pilot BP measurements in the target patient group were used for the sample size calculation. The standard deviation for repeated systolic and diastolic blood pressure measurements in the same patient group was 5.84 and 4.69 mmHg, respectively. A sample size of 16 patients adequately allowed a power of 80% to detect a difference in means across the levels of repeated measures factor equal to 100% of the standard deviation, which is a standardized effect size of 1.0 at a significance level of 0.05 (two tailed). Anticipating a dropout rate of 10%, 18 patients were included in the study.

Continuous variables are presented as mean values accompanied by 95% confidence intervals, while categorical parameters are expressed as percentages of the total cohort. The normality of data distribution was verified using the Shapiro–Wilk test. Differences between modalities were evaluated through repeated-measures analysis of variance (ANOVA), followed by Bonferroni-adjusted post hoc comparisons. A two-tailed *p* value below 0.05 denoted statistical significance. All analyses were performed using Statistica version 13.3 (TIBCO Software Inc., Palo Alto, CA, USA).

## 3. Results

### Interdialytic Blood Pressure by Modality

Mean interdialytic SBP, DBP, and MAP for all five dialysis modalities are summarized in Table 3. The lowest average SBP was observed during HDx (129.1 mmHg; 95% CI 127.3–131.0), whereas the highest was recorded during high-flux HD (133.1 mmHg; 95% CI 131.4–134.9). HDF-post, HDF-pre, and HDF-mixed yielded intermediate SBP values of 133.2 (95% CI 131.4–135.0), 132.3 (95% CI 130.6–134.0), and 131.5 mmHg (95% CI 129.7–133.3), respectively. For DBP, HDx again produced the lowest mean (74.3 mmHg; 95% CI 73.3–75.3), whereas the highest value was noted during HDF-post (77.4 mmHg; 95% CI 76.4–78.4). MAP followed a similar pattern, ranging from 92.6 (95% CI 91.4–93.7) mmHg in HDx to 96.5 (95% CI 95.3–97.7) mmHg in HDF-post.

Repeated-measures ANOVA revealed statistically significant differences across modalities for all BP parameters: SBP (*p* = 0.009), DBP (*p* = 0.0004), and MAP (*p* < 0.001). Bonferroni-adjusted pairwise comparisons (Table 4, Table 5 and Table 6) showed that HDx was associated with significantly lower SBP than both HD (*p* = 0.0196) and HDF-post (*p* = 0.0161). For DBP, HDF-post was significantly higher than HDF-mixed (*p* = 0.0198) and HDx (*p* = 0.00015). Regarding MAP, HDx was significantly lower than HD (*p* = 0.0338), HDF-pre (*p* = 0.0264), and HDF-post (*p* = 0.00002). MAP during HDF-post was also higher than during HDF-mixed (*p* = 0.0198). No other pairwise comparisons reached statistical significance.

## 4. Discussion

BP control remains one of the strongest determinants of cardiovascular outcomes and overall survival among patients on maintenance hemodialysis. Interdialytic BP, which better reflects true ambulatory hemodynamics than pre- or post-dialysis readings, has been shown to correlate more closely with left ventricular hypertrophy, cardiovascular events, and mortality [13]. However, despite its prognostic importance, the influence of dialysis modality on interdialytic BP remains incompletely defined. Conventional high-flux hemodialysis (HD) often leads to intradialytic BP fluctuations and variable volume control, whereas convective techniques such as hemodiafiltration (HDF) and expanded hemodialysis (HDx) have been proposed to offer improved hemodynamic stability [8,9,10,14]. Still, whether these advantages extend into the interdialytic period is uncertain. Most available evidence derives from studies comparing only one or two modalities, typically post-dilution HDF versus HD or HDx versus HD. Large randomized trials such as ESHOL, CONTRAST, and the Turkish OL-HDF study demonstrated that high-volume HDF improves survival relative to HD but found no meaningful differences in BP levels between groups [8,9,10]. Similarly, early evaluations of HDx reported comparable interdialytic BP profiles to high-flux HD, despite improved middle-molecule clearance and reduced inflammatory markers [6,7,12]. A systematic review by Mostovaya et al. confirmed these findings, concluding that the survival benefit of HDF was likely mediated by enhanced solute removal and intradialytic tolerance rather than sustained BP reduction [15]. Thus, the hemodynamic impact of different high-efficiency modalities remains largely unresolved.

Previous research has also highlighted that both absolute BP and its variability across the interdialytic interval influence cardiovascular risk. A meta-analysis by Li et al. showed that higher interdialytic BP variability independently predicted greater all-cause and cardiovascular mortality in over 30,000 dialysis patients [13]. This observation underscores that subtle differences in BP control, even within the normotensive range, may have long-term prognostic implications. Against this background, comparative studies including multiple HDF dilution strategies together with HDx and conventional HD are exceptionally rare. The present investigation addresses this gap by directly evaluating interdialytic BP across all five modalities within the same patient cohort under strictly standardized treatment conditions.

Within this framework, our study demonstrates that HDx consistently yielded significantly lower home BP values compared to high-flux HD and, in particular, post-dilution HDF. Mean interdialytic systolic BP during HDx was approximately 129 (95% CI 127–131) mmHg, versus ~133 mmHg for both high-flux HD and HDF-post. Bonferroni-adjusted pairwise comparisons confirmed significantly lower SBP, DBP, and MAP during HDx compared to these two modalities (*p* < 0.05), while differences with HDF-pre and HDF-mixed were not significant. Although the absolute differences were modest (3–5 mmHg), even small reductions in BP of this magnitude are known to translate into meaningful decreases in cardiovascular risk in both hypertensive and dialysis populations [13]. Potential mechanisms underlying the modest BP advantage observed with HDx include improved removal of middle molecules and protein-bound uremic toxins, attenuation of endothelial dysfunction, and better intradialytic hemodynamic stability [16,17,18]. These physiologic effects may contribute to smoother post-dialysis vascular reactivity and lower interdialytic pressure variability. Taking all this into account, HDx may be particularly advantageous in patients with poorly controlled interdialytic BP despite adherence to pharmacologic therapy and achievement of dry weight. Earlier reports emphasized that volume management and antihypertensive therapy are the principal determinants of BP control in dialysis patients [19,20]. Our results suggest that the appropriate selection of dialysis methods may also have a potential role in this respect. Moreover, the present results extend our prior observations [14], which demonstrated superior intradialytic hemodynamic tolerance with HDx and post-dilution HDF.

Current clinical guidelines support an individualized approach to BP management in dialysis patients. Given the lack of universally accepted BP targets and the potential harms of excessive lowering [21,22], KDIGO and KDOQI recommend focusing on volume status, dry-weight achievement, hemodynamic stability and overall cardiovascular risk rather than pursuing strict numeric thresholds [23,24]. The results of our study are consistent with this approach and indicate the possibility of maintaining hemodynamic stability by choosing the dialysis method, not only during the hemodialysis session procedure but also in the period between dialysis sessions [25].

Our study has certain limitations that should be considered when interpreting the results. First, the small sample size and, consequently, the study’s low power allow for confirmation of significance only for large differences in recorded BP values. The small sample size also precludes multivariate analysis and the influence of other factors on BP, including gender differences. The influence of the patients’ fluid levels on the obtained results cannot be completely ruled out. Maintaining a constant dry weight throughout the study period was to prevent this, but it could also have negative consequences in this regard. Ultrafiltration values (weight gains) during all phases were similar; however, it cannot be ruled out that some patients experienced changes in dry weight and subclinical fluid overload during the study. Perhaps a better solution would have been to assess bioimpedance before each phase of the study. It should also be noted that the study population had relatively well-controlled BP. It would be interesting to see how BP would develop in patients with poorer BP control. Taking all this into account, the results of our study can only be treated as a pilot study. Further large-scale studies in patients with poorly controlled interdialytic BP are warranted to determine the impact of individual high-efficiency dialysis methods on BP control.

## 5. Conclusions

In this randomized cross-over study of stable HD patients, five dialysis modalities—conventional high-flux HD, HDF in pre-, post-, and mixed-dilution configurations, and HDx—were compared under tightly standardized treatment conditions. Although interdialytic BP control was largely comparable across modalities, HDx achieved the lowest systolic, diastolic, and mean arterial pressures. Even small reductions in BP of this magnitude may carry cardiovascular relevance, suggesting that HDx could offer incremental hemodynamic benefit in selected patients with suboptimal BP control.

## Figures and Tables

**Table 1:** Baseline Characteristics of the Study Population (*n* = 16).

Variable	Value
Age (years)	62.6 ± 12.7
Sex (male/female)	10/6
Arterial hypertension	15 (93.75%)
Coronary artery disease (CAD)	5 (31.25%)
Congestive heart failure (CHF)	4 (25%)
History of stroke or transient ischemic attack (TIA)	3 (18.75%)
Diabetes mellitus	9 (56.25%)
On high-efficiency HD prior to study	11 (68.75%)
On different high-efficiency modalities prior to study (HDF-pre, post or HDX)	5 (31.25%)
Number of antihypertensive drugs	
– 1 agent	3 (18.75%)
– 2 agents	6 (37.5%)
– 3 agents	2 (12.5%)
– ≥4 agents	5 (31.25%)
Antihypertensive classes used	
– β-blockers	11 (68.75%)
– α-blockers	5 (31.25%)
– Angiotensin Converting Enzyme inhibitors (ACEi)	4 (25%)
– Angiotensin Receptor Blockers (ARBs)	1 (6.25%)
– Calcium channel blockers (CCB)	10 (62.50%)

**Table 2:** Average Delivered Dialysis and Treatment Parameters Across Modalities.

	High-Flux HD	HDX	PRE-HDF	MIX-HDF	POST-HDF	*p*
Session duration [min]	240	240	240	240	240	NA
Blood flow [mL/min]	300	300	300	300	300	NA
Dialysate flow [mL/min]	500	500	500	500	500	NA
Ultrafiltration volume [mL]	1724 (360)	1664 (364)	1777 (364)	1751 (407)	1902 (411)	0.62 *
Total convection [L]	NA	NA	45.76 (4.582)	50.98 (3.451)	20.68 (1.133)	0.01

Abbreviations: High-flux HD—conventional High-flux hemodialysis; HDF—hemodiafiltration; HDx—expanded hemodialysis. * No statistically significant differences in ultrafiltration volume were observed across modalities (*p* = 0.62, ANOVA).

**Table 3:** Interdialytic Blood Pressure Across Dialysis Modalities (mean (95% confidence intervals), ANOVA *p*-values).

Modality	SBP (mmHg)	DBP (mmHg)	MAP (mmHg)
High-flux HD	133.14 (131.36–134.92)	75.85 (74.86–76.85)	94.95 (93.85–96.05)
HDF-pre	132.29 (130.55–134.03)	75.69 (74.75–76.63)	95.06 (93.93–96.18)
HDF-post	133.22 (131.40–135.03)	77.39 (76.39–78.39)	96.50 (95.31–97.69)
HDF-mixed	131.51 (129.70–133.31)	75.18 (74.15–76.21)	93.96 (92.81–95.10)
HDx	129.12 (127.26–130.98)	74.29 (73.31–75.28)	92.57 (91.43–93.71)

Abbreviations: High-flux HD—conventional High-flux hemodialysis; HDF—hemodiafiltration; HDx—expanded hemodialysis; SBP—systolic blood pressure; DBP—diastolic blood pressure; MAP—mean arterial pressure. Note: HDx was associated with significantly lower SBP, DBP, and MAP compared to high-flux HD and HDF-post (*p* < 0.05, Bonferroni adjusted).

**Table 4:** Bonferroni-Adjusted Pairwise Comparisons for SBP (*p*-values) (ANOVA *p* = 0.009).

	High-Flux HD	HDF-Pre	HDF-Post	HDF-Mixed	HDx
High-flux HD	-	1.0	1.0	1.0	0.020
HDF-pre	1.0	-	1.0	1.0	0.147
HDF-post	1.0	1.0	-	1.0	0.016
HDF-mixed	1.0	1.0	1.0	-	0.665
HDx	0.020	0.147	0.016	0.665	-

Abbreviations: High-flux HD—conventional High-flux hemodialysis; HDF—hemodiafiltration; HDx—expanded hemodialysis.

**Table 5:** Bonferroni-Adjusted Pairwise Comparisons for DBP (*p*-values) (ANOVA *p* = 0.0004).

	High-Flux HD	HDF-Pre	HDF-Post	HDF-Mixed	HDx
High-flux HD	-	1.0	0.315	1.00	0.287
HDF-pre	1.0	-	0.175	1.0	0.499
HDF-post	0.315	0.175	-	0.020	0.000147
HDF-mixed	1.0	1.0	0.020	-	1.0
HDx	0.286	0.498536	0.000147	1.00	-

Abbreviations: High-flux HD—conventional High-flux hemodialysis; HDF—hemodiafiltration; HDx—expanded hemodialysis.

**Table 6:** Bonferroni-Adjusted Pairwise Comparisons for MAP (*p*-values) (ANOVA *p* < 0.001).

	High-Flux HD	HDF-Pre	HDF-Post	HDF-Mixed	HDx
High-flux HD	-	1.0	0.598	1.0	0.034
HDF-pre	1.0	-	0.846	1.0	0.026
HDF-post	0.598	0.846	-	0.020	0.000018
HDF-mixed	1.0	1.0	0.020	-	0.880
HDx	0.034	0.026	0.000018	0.880	-

Abbreviations: High-flux HD—conventional High-flux hemodialysis; HDF—hemodiafiltration; HDx—expanded hemodialysis.

## Data Availability

The data are available from the corresponding authors upon reasonable request.
